# Supplementary figures and images for: Are bivalves susceptible to domestication selection? Using starvation tolerance to test for potential trait changes in eastern oyster larvae
Source: PLoS One. 2020 Jun 30;15(6):e0230222. doi: 10.1371/journal.pone.0230222 (PMC7326227; doi:10.1371/journal.pone.0230222)

**S2 Fig**

**
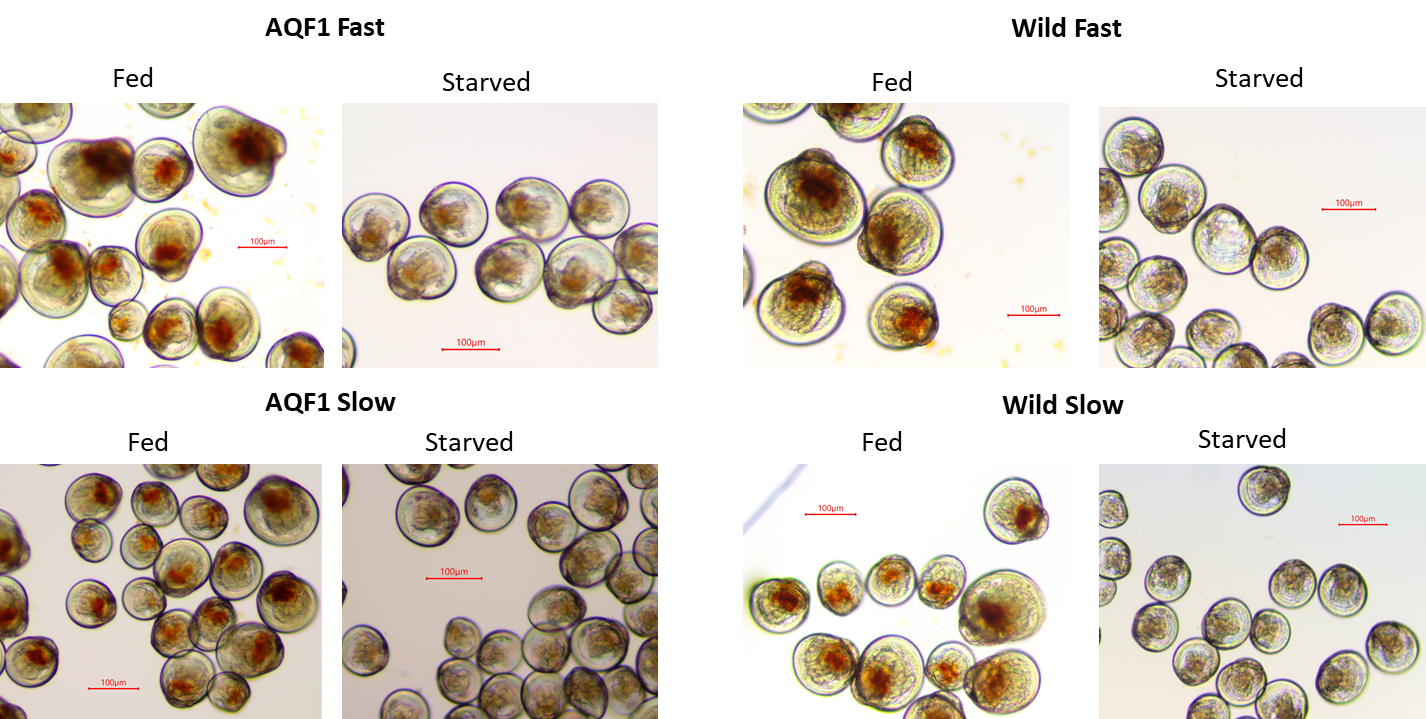
**

Supplement: S2 Fig — (DOCX) [file pone.0230222.s002.docx]

**S3 Fig**

**
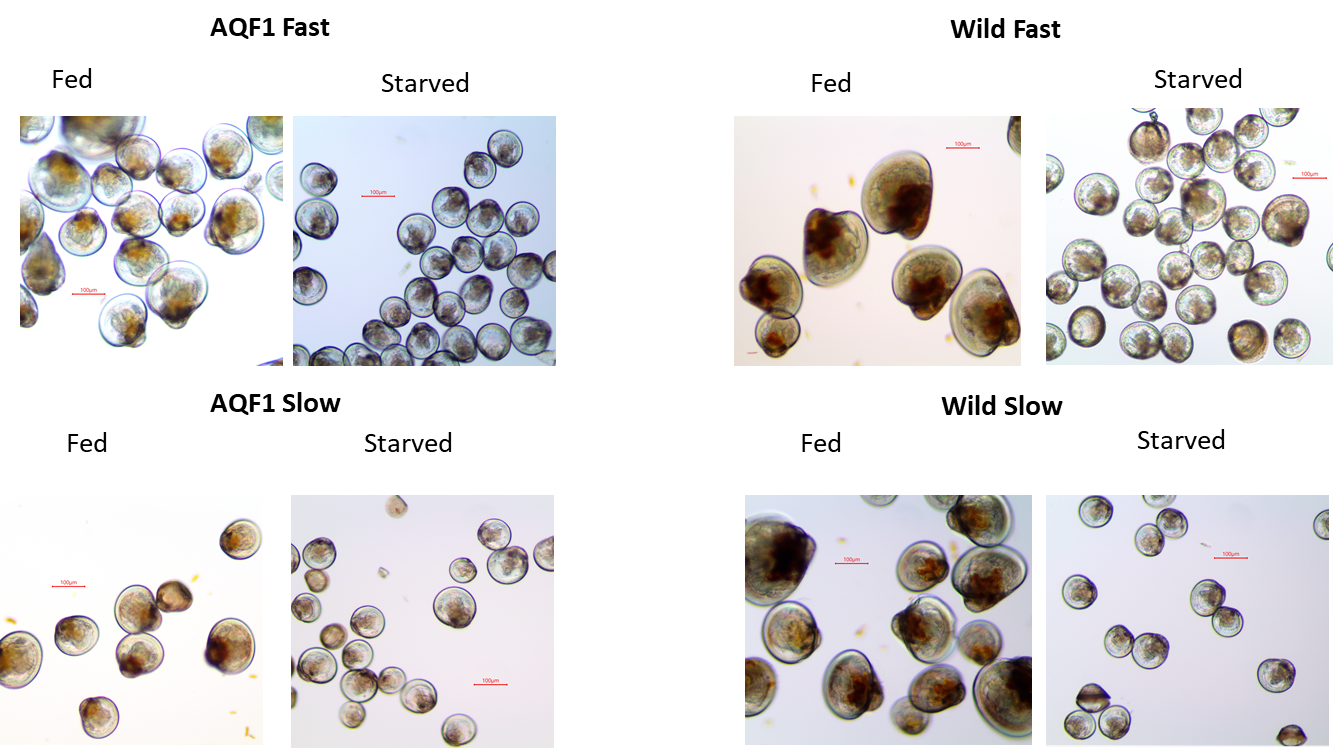
**

Supplement: S3 Fig — (DOCX) [file pone.0230222.s003.docx]

**S4 Fig**


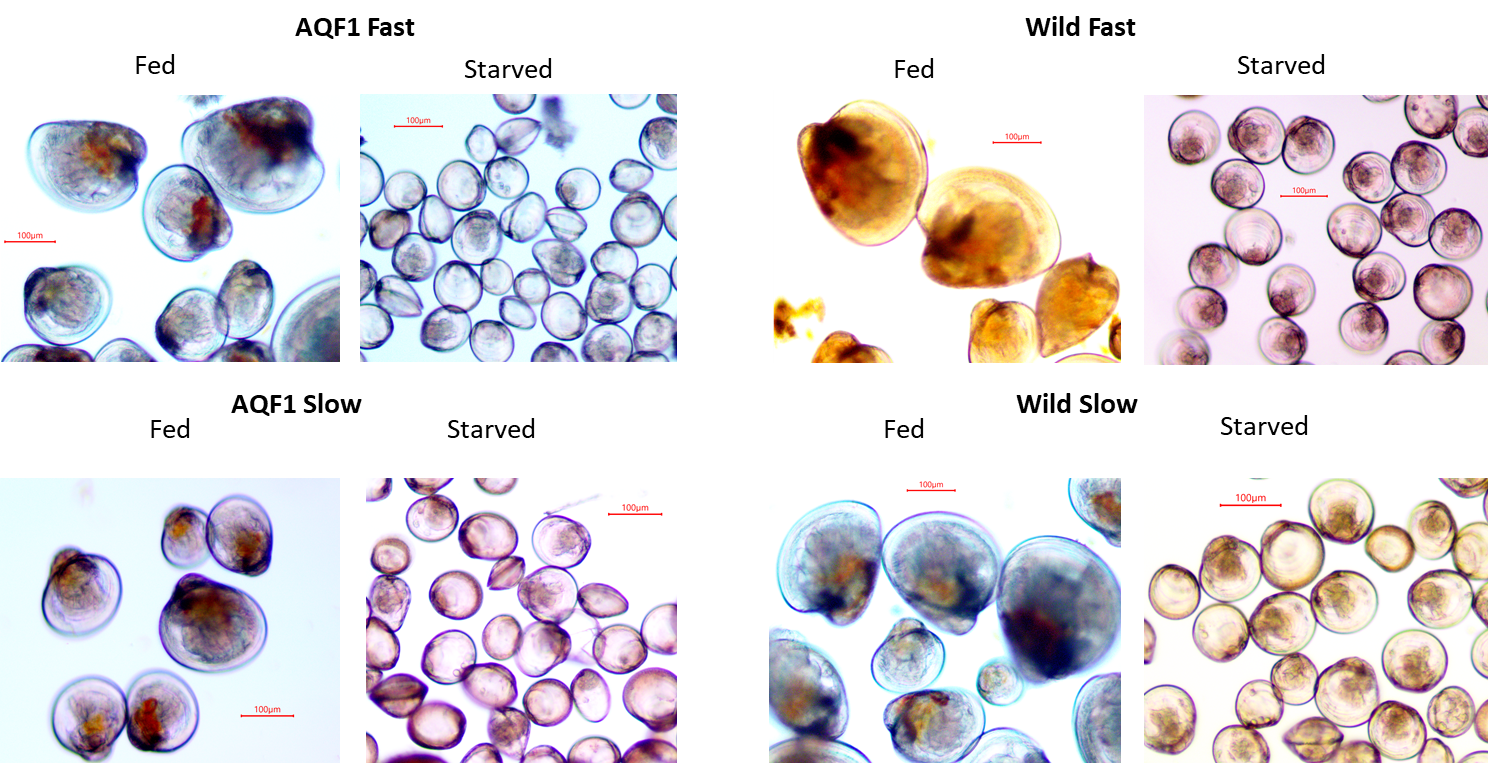

Supplement: S4 Fig — (DOCX) [file pone.0230222.s004.docx]
