# Supplementary material for: Are bivalves susceptible to domestication selection? Using starvation tolerance to test for potential trait changes in eastern oyster larvae
Source: PLoS One. 2020 Jun 30;15(6):e0230222. doi: 10.1371/journal.pone.0230222 (PMC7326227; doi:10.1371/journal.pone.0230222)
Supplement: Attachment — Submitted filename: McFarland et al..pdf [file pone.0230222.s005.pdf]

The question raised by the authors is interesting and their experimental approach and data analyses for the most part fine. They could have perhaps evaluated growth rate in a more quantitative way, and although I don't think it will change the results much, the manner in which they normalized oxygen consumption rate with shell length should be given more thought.

Abstract

A fascinating result was the rapid growth of the starved larvae once feeding was resumed so that by the end of the experiment, Day 29, there was very little difference in shell length among treatments (Fig. 4, S2 Fig). This result could be stressed more in the abstract (line 36). Oxygen consumption rates also recovered (Fig. 5) but there is no mention of the result in the abstract.

Introduction

The introduction provided good background for the study. Perhaps some minor re-organization connecting the hypothesis tested (64-66) and it's basis (111-118). The sentence between 130 – 133 should be deleted as it is repetitive of the previous sentence.

Methods

I am not sure why S1 Fig is not included in the manuscript instead of in supplemental material. I found it useful for understanding the experimental design and suggest it be included in the text. I think in S1, however, there is a mislabel, shouldn't Day 10 actually be Day 17? The starvation period was 10 days, and started on Day 7, correct?

Please explain how oxygen consumption rates were normalized by shell length (244). Although VO_2 will scale to shell length, I would not expect it to be linear.

Results

Survival
Nice photos of the larval guts (e.g., S3 Fig), perhaps include one in the text.

Growth
Why were the actual growth rates of the larvae (i.e., $\mu m\ d^{-1}$) not more quantitatively compared, at least during certain time intervals. For example, once the refed larvae "recovered", did they exhibit a significantly higher growth rate between days 22 and 29 than the control larvae?

I did not follow what was meant by "a steeper decline in size", its sounds as if the larvae were shrinking (348-349).

(369-372) If shell length "showed no significant increase once food was removed" how was it AQF1/Fast exhibited a growth slow down? Also, what is being implied about the observation the starved and fed AQF1/Fast exhibited a slow down in shell growth during the same period. Perhaps just some rewriting here.

Respiration

In addition to my comment about VO_2 normalization with shell length, the unit for respiration on Fig. 5 should be corrected. Also, it is unclear with the wild starved data why n = 2 on the first day and more replicates were run at later dates.

Discussion

430 – I do not understand "release from selection unlimited".
